# A quality improvement intervention to enhance performance and perceived confidence of new internal medicine residents

**DOI:** 10.1080/20009666.2018.1487244

**Published:** 2018-08-23

**Authors:** Ahmed Otokiti, Abdelhaleem Sideeg, Paulisa Ward, Merina Dongol, Mohamed Osman, Oloruntobi Rahaman, Syed Abid

**Affiliations:** Department of Medicine, Harlem Hospital Center, Affiliate of Columbia University of Physicians and Surgeons, NY, USA

**Keywords:** Resident education, July effect, resident orientation, residency orientation, peer orientation, clinical documentation

## Abstract

**Background**: Orientation for new medical residents is challenging due to the diversity of prior experiences and cultural backgrounds and is compounded by a lack of orientation curricula that adequately addresses the needs of the medical residents to allow them to perform their duties in an efficient manner from the start. The beginning of residency training is associated with reduced quality of healthcare widely referred to as the ‘July effect’.

**Objective**: To assess the impact of a peer-led orientation for new interns on (a) self-reported confidence level, (b) improvement in performance of first-year residents in appropriate clinical documentation and efficient discharge procedures and protocols.

**Design/methods**: In June 2016, a hybrid of interactive teaching and simulation exercises was used to teach documentation of critical information, such as discharge medication reconciliation and discharge summary. A handout of an intern guide/manual was also provided. The previous year’s data served as comparison/control data. Comparison data were obtained for both groups from hospital’s utilisation review department.

**Results**: Twenty-one of 23 expected new interns (91%) participated in the intervention. There was a significant decrease in non-compliance for clinical documentation in the intervention group compared to the control group. The self-reported confidence level in the intervention group increased 34%.

**Conclusions**: Such peer-to-peer orientation has the potential to effectively improve appropriate documentation and discharge process by new residents and may help to reduce the ‘July effect’.

## 1. Background

Residency training is a compulsory part of medical education and is required to qualify as an independent medical practitioner in the United States. It is a major component of medical training, and as such, the habits formed during the residency years of practical training leave a lasting footprint on the way a physician will deliver medical care. This ‘rite of passage’ for the medical trainee commences in July and transpires for the next few years depending on the choice of specialty training.

Orientation for new residents (interns) usually begins a few weeks prior to the start date of training on 1 July [1]. Medical school graduates in each residency programme hail from different parts of the country as well as from various countries, with vastly different medical training and cultural backgrounds [2]. This makes it particularly challenging to set a curriculum to provide the necessary orientation that satisfies the needs of this conglomerate of medical residents. Traditionally, orientation for new residents, regardless of the medical specialty, is lumped together and consists of sitting in large auditoriums, listening to day-long lectures regarding the policies and legalities of healthcare delivery [1]. The individual nuances of working in specific departments are left out due to issues of generalisability and time constraints. The lack of familiarity with location and staff in addition to the lack of information and guidance regarding proper documentation, ordering and sending lab tests, and so on leaves residents unacceptably overwhelmed and unprepared to handle their duties, when they start work [3]. Interns are an integral part of healthcare delivery – they work closely with patients and other members of the healthcare delivery team. Adequate training of interns regarding proper documentation is an integral part of the safe and efficient care of patients and is closely related to hospital compliance with regulations and thus hospital reimbursement. The time period corresponding to the beginning of residency training in July is associated with reduced quality of healthcare delivery and patient outcomes [3]. This reduction in quality is well known and documented in the medical literature and is known collectively as the ‘July Effect’ [4–9]. The initiators of this project experienced first-hand the effect of this nationwide inadequacy in training and recognised that deficiencies in orientation translate to added stress not only for residents but also for other members of the collaborative care team. Thus, we aimed to study the effects of a peer-led orientation for new interns utilising a hybrid of interactive teaching and simulation methods. This quality improvement project focuses on teaching interns how to identify critical information, document appropriate findings and decrease ambiguity. The goal of the project was to assess the impact of our intervention on interns in 2016 during the transition months of July–September, compared to performance of previous-year interns during the same period (July–September 2015). This intervention/orientation was conducted by 7 volunteer 2015 interns with guidance from 2 faculty members.

## 2. Design and methods

We obtained an exemption from standardised informed consent for human research study from our institutional review board (IRB). We secured departmental approval for quality improvement activities as required by our hospital policy. This novel quality improvement (Q.I) effort was planned to involve the incoming Internal medicine (I.M) residents and was scheduled to occur in the last week of June 2016, prior to the commencement of residency training on 1 July. Our methodology was based on the principles of Plan-Do-Study-Act (PDSA) 2.

Specific interventions included the following:

1. Interactive teaching and simulation exercises
2. Provision of an intern manual/guide to all participants prepared by graduating first-year residents

The duration of the intervention was 3 hours. First, there was an hour-long interactive session regarding an efficient discharge process. The training included discharge medication reconciliation and discharge summary (DS) documentation. The second hour involved teaching appropriate physician documentation and issues regarding compliance with hospital and New York (NY) state public health mandates. Based on our hospital utilisation review mandates and NY state public health law, all newly admitted patients should be screened for HIV, smoking and domestic violence and counselled appropriately, with adequate documentation of screening and counselling [10].

The third hour was dedicated to the simulation of discharge process with discharge summaries and the medication reconciliation process according our electronic medical record system. The new interns were also taught how to conduct proper handoff during a shift change; issues about the dynamics and workflow in various units were also shared including reflections about challenges faced as interns in different units.

In addition, at the end of the intervention, an intern manual/guide was provided to all participants, which was prepared by current first-year residents. We provided the participant with an electronic and a hard copy of this manual, which contained valuable information to aid in their daily routine. The use of a resident manual has been shown to be highly beneficial to the smooth transition of new residents in previous similar interventions [1]. The manual contained relevant information such as important phone numbers and pager numbers, door access codes for important sites in the hospitals, list of emergency Codes, Do Not Use abbreviation list, instruction on inter-hospital and inter-unit transfer, how to activate the rapid response team (RRT), isolation protocols, discharge summary component, history and physical examination template, and other important information.

## 3. Evaluation of intervention

To assess the impact of our intervention on the confidence level of the interns, we provided a pre- and post-intervention survey. The survey included likert-scale type questions regarding the confidence level related to undertaking various designated responsibilities as new interns. Because of the novelty of our intervention, our literature search did not reveal a validated survey used for similar projects in the past, and therefore the survey that was used is not validated.

These included electronic discharge summary documentation, knowledge of the unacceptable abbreviations in clinical documentation, and medication reconciliation. To objectively assess the impact of our intervention on the 2016 interns during the transition months of July–September 2016, we used performance of previous year (2015) interns during their transition months as control data. Our comparison of the intervention and control groups was based on two domains; a) clinical documentation (comparing the incidence of unacceptable abbreviations used by both groups during the transition months. b) Comparing the level of hospital utilisation and public health infractions committed by both groups during the transition months (HIV screening, smoking screening and counselling, domestic violence screening that were performed).

Comparison data were obtained for both groups from our utilisation review department. Our utilisation review department typically compiles weekly deficiencies in documentation and public health mandates. This helped us compare groups effectively, as the same level of staff members was involved in the data gathering for both groups. A total of 274 charts (control; 154 charts vs intervention; 120 charts) were randomly selected by the utilisation review department staff and reviewed for non-compliance during the transition month.

## 4. Results

As expected, 21 out of the 23 expected new interns (91%), who were able to start the orientation on time, participated in the intervention. There was no significant difference in the demographic characteristics between both groups compared. Both groups included 100% international medical graduates (IMGs), and the intervention group (IG) included 11 males (52%), the control group (CG) 11 (44%; p = 0.57). The average clinical experience of the IG was 4 years (SD: 1.8) compared to CG (mean: 3.32; SD: 1.7; 95% CI: 0.36–1.8; p = 0.34). Other demographic information is shown in Table 1.

**Table 1.** Basic demographic characteristics of intervention and control groups.

Characteristics	Intervention group (n = 21)	Control group (n = 25)	*P* value
Previous U.S. clinical experience > 2 years	6 (28%)	2 (8%)	0.067
All previous clinical experiences > 3 years	12 (57%)	11 (44.0%)	0.37
Total average years of previous clinical experiences	4 ± 1.8	3.32 ± 1.7	0.34
Previous residency training experience	5 (23.8%)	6 (24.0%)	0.98
Previous *EMR experience	13 (61.9%)	12 (48%)	0.35
Previous electronic *DC summary experience	8 (38.1%)	5 (20%)	0.17
Previous QuadraMed system experience	6 (28.6%)	5 (20%)	0.49
Observership/externship at Harlem Hospital	5 (23.8%)	10 (40%)	0.24
Male sex	11 (52.4%)	11(44%)	0.57
*IMGs	21(100%)	25 (100%)	

*DC: discharge; *EMR: electronic medical record; *IMG: international medical graduates.

**Figure 1.**
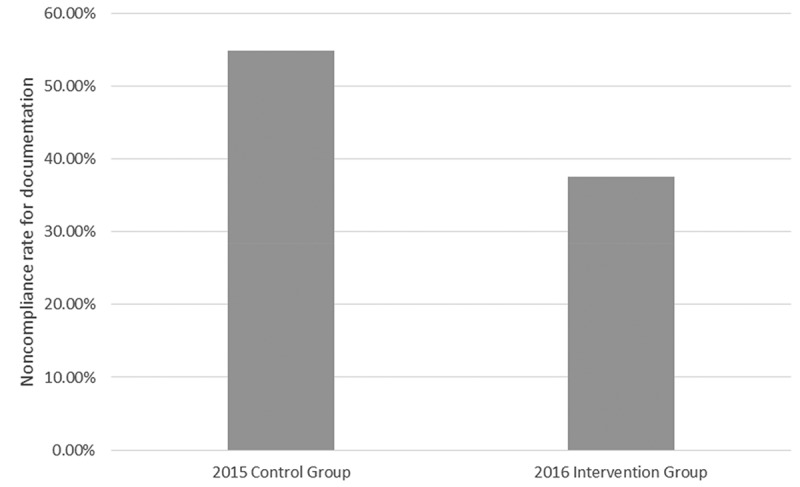
Noncompliance by group during the first 3 months of residency training.

**Figure 2.**
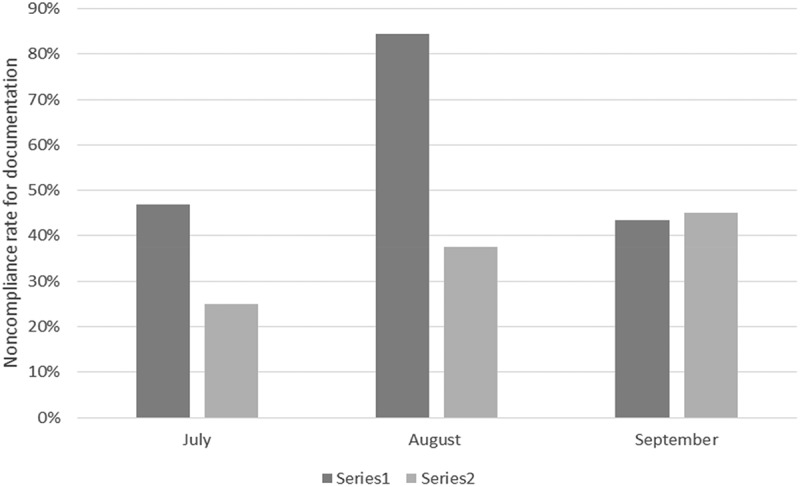
Graph comparing percentage non-compliance per month between the control and intervention group (July–September 2015 vs July–September 2016).

We found that the new interns who had undergone our intervention showed a) mean increase in self-reported confidence level of 34% (95% C.I; range 21%–47%, SD 27.7%) for performance during the transition months regarding overall confidence, discharge summary documentation, efficient discharge process and planning as well as clinical documentation compliance and avoidance of ‘common do-not-use abbreviation lists’. They also demonstrated an objective reduction in clinical documentation non-compliance rates compared to the previous year’s control group. Total non-compliance recorded over the transition month in the control group was 54.8% versus 34.7% in the intervention group (chi-square, P value 0.005) (see Table 2 and Figure 1). The non-compliance rate in both groups was also compared by months. In July, the rate was 46.8% compared to 25%; in August, the rate was 84.4% compared to 37.5%; and in September, the rate was 43.5% compared to 45% in the control versus the intervention group respectively (chi-square, P value 0.001). See Table 3 and Figure 2 for results of month-by-month comparison of both groups.

**Table 2.** Non-compliance in clinical documentation during the first 3 months of residency training.

Intern Group	Non-compliance	Number of charts reviewed
2015 Control group	54.8%	154
2016 Intervention group	37.5%	120

Chi-square test for comparison; P value 0.005.

**Table 3.** Comparison of clinical documentation by month.

Month	2015 Control group			2016 Intervention group		
	Non-compliance count	% Non-compliance rate	Number of charts reviewed	Non-compliance count	% Non-compliance rate	Number of charts reviewed
July	22	46.8%	47	10	25%	40
August	38	84.4%	45	15	37.5%	40
September	27	43.5%	62	18	45%	40

Chi-square test for comparison; P value 0.001.

## 5. Discussion

The ‘July effect’ results in decreased quality of medical care during the months following commencement of residency training, with direct effects on patient care, patient safety, and hospital reimbursement. One of the reasons identified in the literature is a deficiency in the orientation of new interns [1,11]. We set out to study the effects of a well-structured intern to intern peer orientation using interactive learning techniques, simulation modalities, and distribution of intern manual on new interns’ confidence levels and in decreasing errors in documentation during the early months of residency. These modes of delivery have been validated as highly effective in delivering medical education [12]. There was a considerable decrease in noncompliance rates in the intervention group, which seemed to level off by the third month. This is in agreement with Haller and colleagues who found that the risk of undesirable events during the early months of training is independent of clinical experience or residents’ seniority level but is rather more dependent on the familiarity of residents with the particular hospital system [13]. Consequently, it is not surprising that the difference observed in the first and second months between the intervention and control group seemed to level off at the third month due to increasing familiarity with the system by both group members.

The change in overall confidence level was derived by converting the likert scale survey responses to a numerical score. There was a sizable increase in the confidence level of the new interns post-intervention, based on pre- and post-intervention survey administered. Our results showed that our intervention may have increased new interns’ skills in clinical documentation compared to the previous year’s interns while also boosting their confidence to perform well. We believe a similar intervention can be replicated with appropriate modifications to meet the requirement of other residency programmes in different specialties. If adopted nationwide, we believe it may help to reduce the ‘July effect’.

Generalisability of our intervention was limited by a small sample size. It also involved 100% IMGs which may also limit the generalisability of our result. Variations in the composition of residents and the location of the healthcare center (inner city vs. rural area) are factors that may impact the generalisability of our results.

## 6. Conclusions

Our intervention resulted in improvement in clinical documentation and the discharge process while increasing confidence levels of the new interns. More and similar interventions are needed to examine the impact of such peer-to-peer orientation on quality and efficiency of care delivered by new interns.
